# The use of advanced imaging in guiding the further investigation and treatment of primary prostate cancer

**DOI:** 10.1186/s40644-022-00481-3

**Published:** 2022-09-03

**Authors:** Heying Duan, Andrei Iagaru

**Affiliations:** Department of Radiology, Division of Nuclear Medicine and Molecular Imaging, Stanford University, Stanford, CA USA

**Keywords:** Image-guided, mpMRI, ^68^ Ga-RM2, ^68^ Ga-PSMA, PET, Prostate Cancer

## Abstract

In the era of precision medicine, oncological imaging techniques are advancing at a rapid pace, particularly molecular imaging with promising new targets for prostate cancer (PC) such as gastrin releasing peptide receptors (GRPR) along the established and indispensable prostate specific membrane antigen (PSMA). As PC is characterized by heterogenous tumor biology ranging from indolent to aggressive disease, distinguishing clinically significant tumors from indolent disease is critical. Multiparametric MRI- and PET-targeted prostate biopsies mitigate the shortcomings and risks of standard systematic template biopsy by identifying more significant cancers.

Focal treatment for localized disease is a minimally invasive approach that targets the index tumor – the lesion of the highest grade – while sparing the surrounding healthy tissue. Real-time MRI-guidance and thermal control with MR-thermometry, improves treatment accuracy and results in lower rates of functional side effects. PET imaging could be an useful tool to assess response to treatment compared to invasive prostate biopsies.

In this comprehensive review, we focus on the image-guided detection and treatment of localized primary prostate cancer, its current status and future perspectives.

## Introduction

Prostate cancer (PC) is the most frequent non-cutaneous cancer in the US with one in every eight men diagnosed with PC during their lifetime [1]. Worldwide, PC is the second most frequent malignancy with an estimated 1.4 million new cases and 375,000 deaths ranking as the fifth leading cause of cancer deaths among men in 2020 [2]. Due to the high volume and population affected, PC is considered a global health problem. Screening for serum prostate specific antigen (PSA) has dramatically increased the diagnosis of PC; however, many are low-grade, clinically non-significant cancers, leading to overdiagnosis and overtreatment. This resulted in an increase of therapy-associated side effects such as erectile dysfunction and incontinence, and of the economic burden on the healthcare system. *To screen, or not to screen, that is the question* or better put, the dilemma. There is a clinical need for faster and more accurate ways to identify clinically significant PC in order to reduce the harms of screening while maintaining the benefits.

The underlying tumor biology of PC is heterogenous and on a spectrum with reclassification over time, spanning from indolent disease, characterized by Gleason score 3 + 3, to clinically significant, aggressive cancer with Gleason score ≥ 3 + 4. It is not only important to differentiate between non-significant and significant cancers, but also whether the disease is localized or metastasized and if so, to what extent. Accurate detection of suspected PC is crucial to direct subsequent patient management. While low-risk and some subsets of intermediate-risk, indolent disease is typically cared for with active surveillance [3, 4], aggressive cancers require therapy. The treatment options are multifaceted and include prostatectomy, radiation therapy, hormonal therapy, chemotherapy, or a combination of these [5–7]. While a whole-gland treatment approach shows good oncological results, it may also have life-altering side effects such as incontinence, impotence, and infection [8]. As the majority of PC are localized within the prostate gland [9], minimal invasive local treatment approaches have been gaining in interest and popularity as the adverse events reported are low while having good oncological outcome [10].

The role of imaging is indispensable and used not only to detect PC, but also to assess tumor volume and extraprostatic extension, and to guide targeted biopsy and focal treatment. Multiparametric magnetic resonance imaging (mpMRI) has become the gold standard in staging PC as it has high sensitivity; mpMRI-targeted biopsies find more clinically significant and less insignificant tumors compared to systematic transrectal ultrasound (TRUS)-guided biopsies [11]. It is increasingly used for treatment planning and guidance of focal therapy in localized PC. However, there are limitations to mpMRI as clinically significant PC may be missed [12–16].

Molecular imaging with positron emission tomography (PET) combined with computed tomography (CT) or MRI provides anatomical and biological information of the whole body. Especially PET/MRI with its high soft tissue contrast is well-suited for staging PC. The functional information is obtained from agents that target different receptors on the PC cell. The most widely used radiopharmaceutical targets the prostate specific membrane antigen (PSMA) which is overexpressed in 90% of PC [17]. Another promising target is the gastrin releasing peptide receptor (GRPR) which is highly overexpressed in several cancers including PC with favorable characteristics for initial staging of PC [18–21].

In this review article, we focus on the *status quo* of image-guided, targeted prostate biopsies and focal treatments for localized primary PC using mpMRI and PET with gallium-68 (^68^ Ga) radiolabeled PSMA- and GRPR-targeting radiopharmaceuticals and give an outlook into future directions for image-guided interventions.

### Image-guided prostate biopsy

PC is most often multifocal, arising in 80%–85% of cases from the peripheral zone, 10%–15% from the transition zone, and 5%–10% from the central zone [9]. The index lesion is the highest-grade tumor which drives subsequent management and clinical outcomes [22, 23]. The role of imaging at initial staging is to distinguish clinically significant from indolent disease and to guide targeted biopsy of the index tumor.

### mpMRI-guided biopsy

Traditionally, patients with elevated PSA undergo TRUS-guided biopsy using a non-targeted, systematic 12-core approach to sample the whole prostate. This technique leads to overdiagnosis of insignificant disease while missing clinically significant cancers. Especially cancers located anteriorly are difficult to reach, and are not always part of the biopsy template [24]. Furthermore, TRUS-guided biopsies are associated with more serious complications requiring hospital admission [25, 26].

mpMRI consists of 3 phases: T2 weighted imaging (T2WI) for anatomical, diffusion weighted images (DWI) for biological, and dynamic contrast-enhanced (DCE) imaging for vascular information. The added conspicuity of suspected lesions seen in these specific phases, interpreted using the Prostate Imaging Reporting and Data System (PI-RADS) score [27, 28], together with lesion volume, has increased the sensitivity and specificity for clinically significant cancers to a pooled 89% and 73%, respectively [29].

Multiple clinical trials investigated whether mpMRI can accurately stratify clinically significant to non-significant PC, and compared mpMRI-guided to standard TRUS-guided biopsy. The PROMIS study showed that mpMRI had significantly better sensitivity and negative predictive value for significant disease, and when used as a triage test in biopsy naïve patients, avoided unnecessary biopsies in 27% [30]. The PRECISION trial randomized 500 biopsy naïve men for mpMRI-targeted or systematic biopsy and showed similar results with mpMRI increasing the detection rate of clinically significant PC from 26 to 38%, while reducing the detection of insignificant disease from 22 to 9% [31]. In a head-to-head comparison of mpMRI- and TRUS-guided biopsy, the 4 M trial found identical detection rates of significant disease, but mpMRI detected fewer insignificant cancers and reduced biopsies by nearly 50% [13]. A combined mpMRI- and TRUS-guided biopsy approach, however, showed the best detection rate of clinically significant PC as 7% were missed when mpMRI-guided biopsy was performed alone. The MRI FIRST trial reported a similar miss rate of 5% for significant disease [14] while the TRIO study showed 9% misclassification for mpMRI-targeted biopsy [32]. These miss rates beg the need for other imaging modalities.

In a meta-analysis, mpMRI-targeted and systematic biopsies were compared to histopathology after prostatectomy: a tumor upgrade was found in 23% for mpMRI-targeted versus 43% for systematic biopsy [33]. The PRECISE trial confirmed previous findings that a third of patients (37%) had a negative mpMRI and thus avoided biopsy [34]. mpMRI-targeted biopsy is non-inferior to systematic TRUS-guided biopsy in detecting clinically significant PC, however, the difference was lower (5.2%) than in the PRECISION trial (12%) suggesting that a combined approach might improve detection rates of significant disease.

MRI allows for in-bore or in-gantry biopsy where the procedure is performed in the MRI machine under real-time image guidance with the possibility for immediate correction of a suboptimal needle trajectory. A large case series including 554 patients undergoing in-bore MRI-targeted biopsy showed an overall detection rate of 80% for PC, and 55% for clinically significant disease, even in small, ≤ 5 mm tumors [35]. In patients with prior negative biopsy, PC was found in 60%, of which 80% were significant disease whereas the majority was located anterior in the prostate where TRUS-guided biopsy has known limitations. Half of the active surveillance cohort were upgraded after in-bore biopsy. In a comparison of in-bore MRI-guided and MRI-TRUS fusion-targeted biopsies, in-gantry biopsy detected more clinically significant (61%) and fewer insignificant (11%) PC lesions than MRI-TRUS fusion (41% and 18%, respectively) [36]. These results were validated by recently published studies focused on PI-RADS 4 and 5 lesions [37, 38]. Despite the growing evidence that in-bore MRI-targeted biopsies can accurately detect more significant disease, its use has been limited by the higher costs as MR-compatible instruments, access to scanner, and scanning time are required as well as the learning curve for the interventionist.

As the demand for mpMRI increases, faster imaging techniques are needed. In the updated PI-RADS classification [39], DCE was rated less significant, hence biparametric MRI (bpMRI) without the DCE phase might be useful. Several studies compared mpMRI to bpMRI and to ‘fast’ bpMRI consisting of only 1 plane versus the regular 3 planes. Similar detection rates were seen whereas bpMRI was non-inferior to mpMRI [40–43]. However, and this applies to mpMRI as well, the PROMIS and PRECISION trials have shown a slight discrepancy in expertise expressed as moderate agreement between the site-readers and central expert-readers despite the use of a standardized, PI-RADSv2 scoring system. Therefore, omitting the DCE phase may increase uncertainty in less experienced radiologists. mpMRI and bpMRI are only as good as the used equipment and the radiologist interpreting the images.

Despite these studies showing the overall better performance of mpMRI-targeted prostate biopsies, it is optional to add mpMRI-targeted to TRUS-guided prostate biopsy in biopsy-naïve patients according to the current guidelines of the National Comprehensive Cancer Network (NCCN). However, in patients with prior negative prostate biopsy, mpMRI-targeted biopsy is recommended for repeat biopsy [44].

Advancements in new applications will further optimize image-guided interventions. An innovative way to integrate prior imaging into real-time biopsy is augmented reality. Through ‘smart glasses’, prior mpMRI was matched with real-time TRUS images at standard template fusion biopsy [45]. This approach yielded in a higher PC detection rate of 46% than standard biopsy at 27%. These encouraging results warrant more studies involving the rapidly developing field of novel technology.

### PET-guided biopsies

**Fig. 1.**
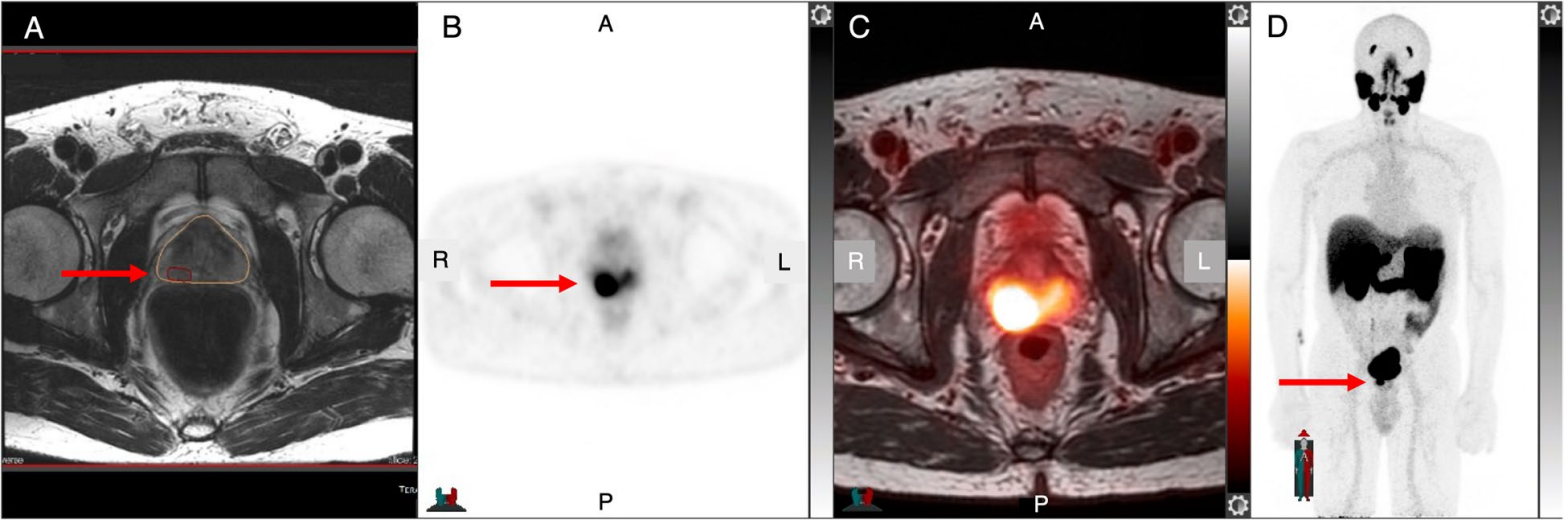
48-year-old man presents with PSA 11.30 ng/mL and PSA density 0.31 ng/mL^2^ for targeted prostate biopsy: mpMRI **A** shows a PI-RADS 4 lesion in the right lateral base whereas ^68^ Ga-PSMA11 axial PET **B**, axial fused PET/MRI **C**, and maximum intensity projection (MIP) **D** reveal a larger tumor volume. Subsequent PET-targeted biopsy resulted in a Gleason score 4 + 3 prostate cancer

As mpMRI misses 5–10% of clinically significant PC, especially in the ‘blind spots’ (transition and central zones) [46], and underestimates the actual tumor volume by up to 3 times (Fig. 1) [47], other modalities are needed to delineate all aggressive lesions. PSMA is a transmembrane protein which is overexpressed in PC [48]; PSMA-targeting compounds have shown high sensitivity and specificity at staging, treatment response evaluation, and biochemical recurrence. Retrospective studies comparing ^68^ Ga-PSMA11 PET/CT [49, 50] or PET/MRI [51] and mpMRI to post-prostatectomy histopathology demonstrated that ^68^ Ga-PSMA PET was superior than mpMRI alone, especially in the detection of additional and smaller cancer lesions. However, smaller lesion with high uptake on PET might lead to overestimation tumor volume due to partial volume effect.

The first prospective study on the feasibility of ^68^ Ga-PSMA617 PET/CT-guided biopsy evaluated men with prior negative standard biopsy but persistent clinical suspicion for PC [52]. On a per patient level, PET/CT-US-guided biopsy detected significant PC in 39% versus 32% for TRUS-guided, whereas a combined approach again increased detection to 67% in patients with positive ^68^ Ga-PSMA617 PET while neither targeted nor systematic biopsy detected clinically significant PC when ^68^ Ga-PSMA617 PET was negative. A prospective, single-center study compared ^68^ Ga-PSMA11 PET/MRI-guided to standard template biopsy in biopsy naïve patients: while PET/MRI showed a 90% accuracy for significant PC with high sensitivity (96%) and specificity (81%), PET-guided biopsy showed a decreased accuracy rate of 71% [53]. The work-up revealed that some of the PET-targeted lesions were missed suggesting that additional perilesional biopsy cores could improve accuracy. This was also observed in the 4 M trial comparing TRUS- to mpMRI-guided biopsy and was related to sampling errors due to spatial heterogeneity of the tumor [13]. When correlated to final post-prostatectomy pathology, ‘false positive’ lesions at biopsy were in fact in 89% PC and proved only in 11% to be benign. As for ‘false negative’ lesions at biopsy, 50% were PC on final histology, 33% did not undergo surgery, and 17% were PC without PSMA expression on immunohistochemistry. This reflects the reported overall rate of PSMA-negative lesions of 5–10% [54, 55]. Lastly, omitting biopsy in patients with negative ^68^ Ga-PSMA11 PET/MRI would have reduced biopsy rate by 33% with missing significant disease in only one patient (7%) who had a PI-RADS 5 lesion on mpMRI.

The results of the prospective multicenter PRIMARY trial evaluating the added value of a pelvic PSMA PET/CT to standard mpMRI showed that a combined approach of PET/CT- and mpMRI-guided biopsy improved sensitivity (97% versus 83%) and negative predictive value (91% versus 72%) for clinically significant PC as compared to mpMRI alone [56]. Nineteen percent of men were negative in both modalities and could have avoided biopsy indicating that a combination of PET and mpMRI act as a better triage tool to discriminate between clinically significant and indolent disease than either one alone.

^68^ Ga-PSMA11 PET/CT was used to guide prostate biopsy through the gluteal muscle and identified clinically significant PC in 80% versus 25% by standard TRUS-guided biopsy [57]. The detection rate was significantly higher in ^68^ Ga-PSMA11 positive than negative scans whereas by omitting biopsy in PET negative patients, 6% of clinically significant cancers would have been missed. Therefore, the authors hypothesize, PET negative patients might benefit from active surveillance rather than excessive biopsies. This transgluteal biopsy technique had no adverse events whereas in the TRUS-guided group, hematuria, urine retention, and infection were observed.

Fluorine-18 (^18^F)-radiolabeled PSMA ligands benefit from the more favorable physical properties: the lower kinetic energy results in a higher spatial resolution, and the longer half-life (110 versus 68 min) allows for a better tumor to background ratio in delayed imaging when compared to ^68^ Ga. Both, ^68^ Ga-PSMA11 and ^18^F-DCFPyL have been approved by the US Food and Drug Administration in 2021. The DeTeCT trial evaluated the performance of ^18^F-DCFPyL PET/CT in identifying primary PC and employed a prostate-mapping model to predict the potential outcome of ^18^F-DCFPyL PET/CT-targeted biopsy [58]. The detection rate of clinically significant PC was forecasted to be 93% with identification of the index lesion in 87%. Consequently, a pilot study investigated the feasibility of ^18^F-DCFPyL PET/CT- or PET/MRI-US-guided prostate biopsy [59]: The detection rate of significant disease was slightly higher for PET/CT-US- at 88% versus 83% for PET/MRI-US-guided biopsies. A small subgroup underwent both ^18^F-DCFPyL PET/CT and PET/MRI whereas MRI was able to confirm PET positive lesions as suspicious, or as benign which was validated by subsequent biopsy.

**Fig. 2.**
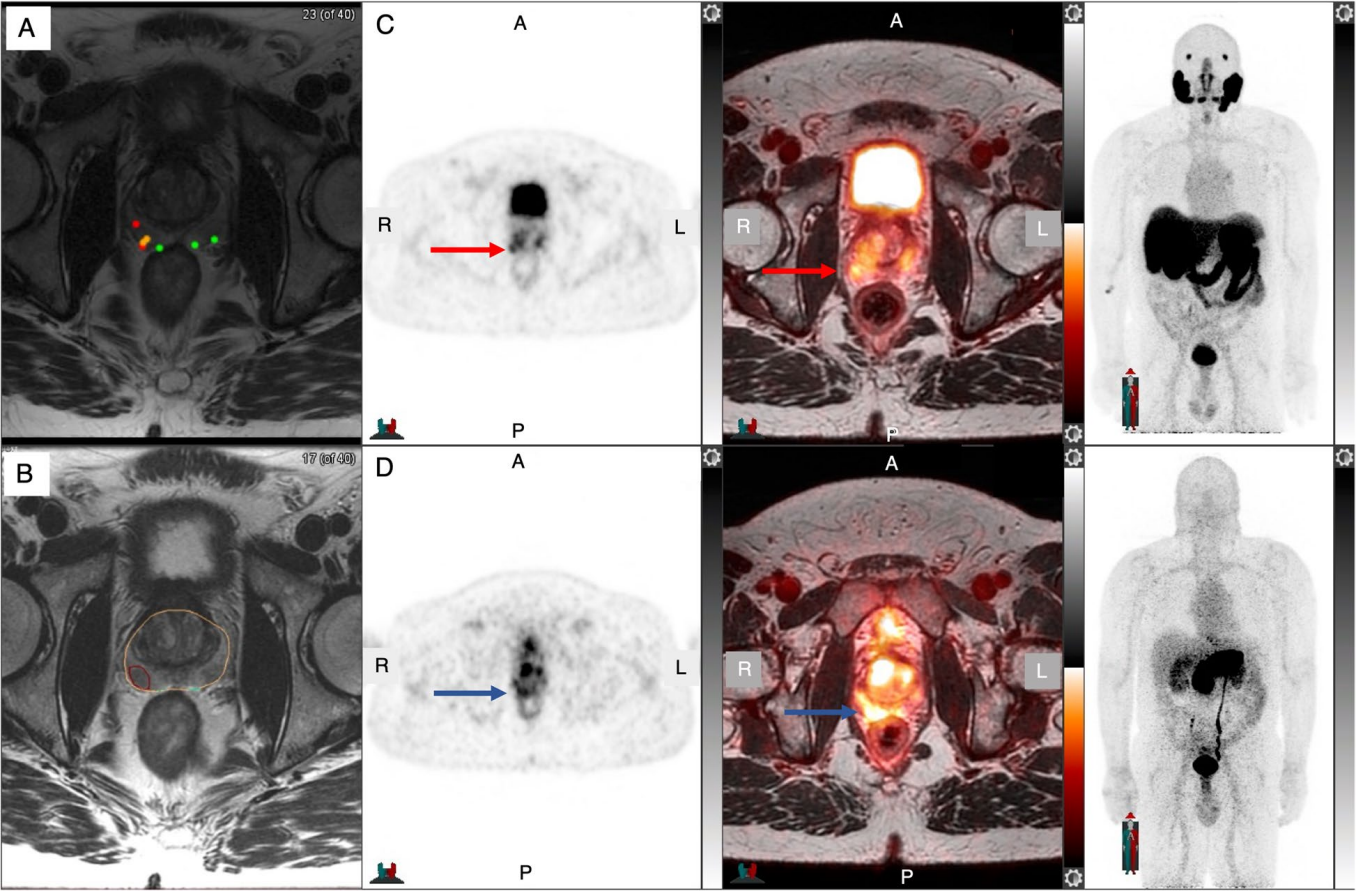
62-year-old man with PSA 7.0 ng/mL and PSA density 0.24 ng/mL^2^. MRI shows a PI-RADS 4 lesion in the right lateral base with color coded needle tracks from biopsy; green-benign, yellow-Gleason score 3 + 3, red-Gleason score 3 + 4 or higher **A**, and target tumor volume **B**. ^68^ Ga-PSMA11 **C** and ^68^ Ga-RM2 **D** axial PET, axial fused PET/MRI, and MIP show congruent focal uptake in the right prostate lesion. The lesion was treated with HIFU; resolution on both ^68^ Ga-PSMA11 and ^68^ Ga-RM2 PET/MRI was seen 6 months after treatment

Lack of specificity of PSMA leads to false positives, while lack of expression of PSMA leads to false negatives [60–66], while up to 10% of PC do not express PSMA [55]. Consequently, other targets were developed. GRPR is highly overexpressed in several cancers including PC, especially in earlier stages, making it an attractive target for initial staging [18–21]. In a large pilot study including 112 men with suspected PC, ^68^ Ga-PSMA617-, the GRPR-targeting ^68^ Ga-RM26 PET/CT-, and mpMRI-targeted prostate biopsy were compared to standard template biopsy [67]. The dual-tracer approach of ^68^ Ga-PSMA617- and ^68^ Ga-RM26-targeted biopsy showed the highest detection rate of 77% without missing any significant cancers. Single ^68^ Ga-PSMA617- and ^68^ Ga-RM26-guided detection rates were at 70% and 56%, respectively, whereas mpMRI-guided and standard biopsy were comparably low at 36% and 35%, respectively. Our group compared in a pilot study ^68^ Ga-PSMA11- and ^68^ Ga-RM2-PET-targeted biopsy in a selected cohort with negative or equivocal mpMRI and/or negative biopsy, but persistent clinical suspicion for PC (Fig. 2) [68]. The preliminary results showed that ^68^ Ga-RM2 was able to detect all clinically significant and non-significant PC with a high sensitivity of 83% whereas ^68^ Ga-PSMA11 showed a lower sensitivity of 63% and missed significant disease in 29%. The low PSMA detection rate is comparable to reported rates for this specific clinical scenario and might reflect a change in tumor biology [52]. PSMA and GRPR expression have been reported as complementary [69, 70], and as GRPR is particularly overexpressed in earlier stages of PC [18], GRPR-targeting radiopharmaceuticals may be more suitable in this specific clinical scenario.

PET-guided biopsies can also be performed in-bore: A recently published study including 78 patients showed that the use of a robotic arm to assist with ^68^ Ga-PSMA11 PET/CT-targeted transgluteal prostatic biopsy is not only safe but detected PC in 96% of patients whereas 44% were clinically significant [71].

mpMRI- and PET-targeted prostate biopsy have shown to have a higher detection rate for clinically significant PC than standard template biopsy. Further prospective studies are needed to answer the central question whether targeted biopsy can retire systematic biopsy. As PET with its added costs may not be easily added to the diagnostic algorithm of PC, it may become relevant when mpMRI is negative or equivocal, or MRI-guided biopsy is negative.

PET-guided prostate biopsy is currently not mentioned in any guidelines. However, the updated NCCN guidelines now recommend PSMA-PET as first-line imaging tool as it is ‘equally effective, if not more effective than conventional imaging’ at both initial staging and biochemical recurrence [72].

### Image-guided focal treatments

As up to 95% of newly diagnosed PC are localized within the prostate gland and are nonmetastatic [9], image-guided, targeted, local treatments might be an advantageous option. Although PC presents in most cases multifocally, the ablation of the index tumor, which is the main driver for morbidity, can lead to tumor control. Minimally invasive, local treatments for PC employ ablation with heat such as high intensity focused ultrasound (HIFU) and focal laser ablation (FLA), or freezing with needle cryoprobes amongst a vast array of different other focal treatments. These procedures aim at the index lesion while sparing the surrounding healthy tissue including the urethra and bladder, the neurovascular bundles, and the rectum. Local therapy approaches preserve continence in 98% and sexual function in 90% of patients compared to whole-gland treatment, leading to only minimal impact on quality of life. [10, 73]. Cancer control over 8 years were similar in a propensity score matched cohort of 335 patients undergoing radical prostatectomy and 501 patients receiving focal therapy [74]. However, long-term data from randomized controlled trials are sparse.

### mpMRI-guided focal treatment

Therapy planning includes identification of the index lesion, assessment of tumor volume and extent of disease. As tumor volume is known to be underestimated by mpMRI, especially tumors with high Gleason scores and small lesions [75], a 20% larger treatment zone than the actual index lesion on mpMRI combined with a 9 mm margin around the lesion has been proposed to ensure treatment of the entire tumor [76, 77].

### HIFU

HIFU is a noninvasive local treatment that employs high-frequency sonographic waves to deliver focal, high energy to the tumor, reaching a temperature of approximately 80 °C causing thermal, mechanical, and tissue effects leading to coagulation necrosis [78]. The probe is commonly placed transrectally and heat is applied for seconds followed by a cooling period to protect rectal mucosa. After HIFU, the lesion may appear cystic with increased T2 signal intensity and hypovascular on contrast-enhanced MRI [79].

Most studies with long-term follow-up data use mpMRI-TRUS fusion HIFU. These studies have shown promising results with low in-field recurrence, i.e., within the treatment zone, of 13% and low urinary incontinence rate of 2% at 5-year follow-up [80]. Erectile dysfunction was seen in 10% and increased insignificantly after repeat HIFU to 21% [73]. Re-treatment with HIFU was necessary for the majority of patients after initial HIFU within a follow-up period of 8 years [81].

In-bore HIFU uses real-time MR imaging to track and guide the HIFU probe and leverages MR thermometry for real-time heat mapping to ensure a precise ablation of the tumor [82]. A first feasibility study showed in 14 patients with low-volume and low-grade PC that this technique is feasible and safe with only transient insignificant deterioration in urinary and sexual function, which resolved within 3 months after HIFU. At 6-month biopsy, 7% of patients showed persistent significant in-field disease, and 17% at 24-month biopsy. As this was a pilot study on safety and feasibility, patients with insignificant disease were also included. Another pilot study evaluated 8 men with low- to intermediate-risk PC and found 60% of treated lesions cancer free at 6-month biopsy while preserving quality of life [83]. In the subsequent prospective phase II trial including 44 men with significant Gleason grade 2 and 3 PC, 93% were free of clinically significant PC at 5-month biopsy while 7% showed persistent disease in the treatment area [84]. Concordant with previous studies, urinary and sexual function showed an insignificant decline following HIFU but resolved at 5-month follow-up. Interestingly, no functional changes were reported for treatment volumes where the neurovascular bundle, urethra, or both were included or spared. These results are encouraging and studies showing long-term data are awaited.

### Transurethral ultrasound ablation

Transurethral Ultrasound Ablation (TULSA) is performed in-bore where the HIFU probe is placed through the urethra. The reported advantage over transrectal HIFU is that it is faster and allows for a more accurate coagulation of the index tumor [85]. In feasibility studies, TULSA was well tolerated by all patients and safe to treat the whole prostate gland with a reduction of viable prostate volume by 88% 12 months after TULSA [86, 87]. A prospective multicenter trial, including 115 men with localized, low- to intermediate-risk PC, reported an average ablation delivery time of 50 min for whole gland TULSA with 98% thermal coverage of the target volume [88]. At 12-month follow-up, treatment failure for any disease was seen in 35%, and for clinically significant disease in 21%. These rates are comparable to biopsy results after external beam radiation therapy including stereotactic body radiation [89]. Functional outcome was comparable to HIFU with preservation of potency in 75% and only transient urinary dysfunction. There is still paucity in data, especially for long-term outcome.

### Focal laser ablation

FLA delivers thermal laser energy through optical fibers that are placed either transrectally or transperineally under in-bore MRI- and thermometry-guidance. Data from a phase I study including 9 low-grade PC patients were promising with 78% showing no evidence of PC while 22% were downstaged to indolent disease [90]. The subsequent phase II study included 27 men with low- to intermediate-risk PC and showed a local recurrence rate of 11% after 1 year [91]. A study involving 8 men with intermediate-risk PC had a recurrence rate in the treated zone of 25% at 6-month follow-up [92]. All these studies reported no deterioration of functional outcome and good tolerance. The largest trial hitherto included 120 patients with low- to intermediate-risk PC; 17% of patients required retreatment at 1-year follow-up. No deterioration in urinary or sexual functional outcome were seen [93]. In a 3-year follow-up after FLA including 15 patients, 47% showed local recurrence whereas salvage treatment in form of repeat FLA and radical prostatectomy were performed in 27% [94]. A recently published study reported 5-year outcomes after FLA in 30 patients of which 83% remained free from failure, defined as prevention of whole gland or systemic treatment, PC metastases, or death; 40% developed in-field recurrence and required repeat ablation. [95]. Despite encouraging early results, these two studies with longer follow-up showed a decline in sexual function as well as a recurrence rate in nearly half of the cohort. One possible explanation for the high relapse rate might be related to the commonly use of a single laser fiber per treatment despite that multiple ablations may be required, potentially leading to undertreatment.

### Cryotherapy

Cryotherapy induces cell apoptosis through repeat freezing and thawing of the PC lesion via transperineally or transrectally inserted cryoneedles under MRI guidance. It can be used for whole or partial gland treatment. Most studies used mpMRI-TRUS cognitive fusion for targeting the index tumor. A recent study reported 10-year oncologic outcome data of 121 men undergoing focal cryotherapy; 65% had low-, 33% intermediate-, and 2% high-risk disease [96]. Despite high overall survival (97%), half of the cohort required subsequent radical therapy. Therefore, compared to active surveillance, adding no significant oncological benefit. Up to 34% erectile dysfunction was reported – the highest within focal treatments – while urinary incontinence rates were comparably low with 5% [97–102].

Data on the performance and long-term oncological and functional outcome of MRI-guided focal therapies for PC are mostly single center, cohort studies and often retrospective which limits a direct comparison of the techniques. Prospective head-to-head comparison or randomized, controlled trials are needed to evaluate the benefits of each local treatment approach.

The current NCCN guidelines only recommend HIFU and cryosurgery with a category 2B evidence (based upon lower-level evidence, but NCCN consensus that the intervention is appropriate) [72]. However, cryosurgery *per definitionem* refers to performing cryotherapy using an open, surgical approach. All other local therapies are not recommended as routine primary therapy due to lack of long-term data.

### PET-guided focal therapies

Using PET imaging to guide focal treatment of PC has not been explored yet. An arena where PET might have a big impact is in the treatment response assessment and generally, post-therapy monitoring. This is an area of unmet clinical need as there are no non-invasive, validated methods or consensus. PSA is an unreliable marker as it falls to a variable nadir due to continued PSA production in the residual gland. Imaging with mpMRI is impeded by post-therapeutical signal alterations such as central necrosis, scar tissue formation or focal hemorrhage which decreases specificity [103, 104]. Prostate biopsy is currently the most accurate tool to evaluate response to therapy with its associated risks.

**Fig. 3.**
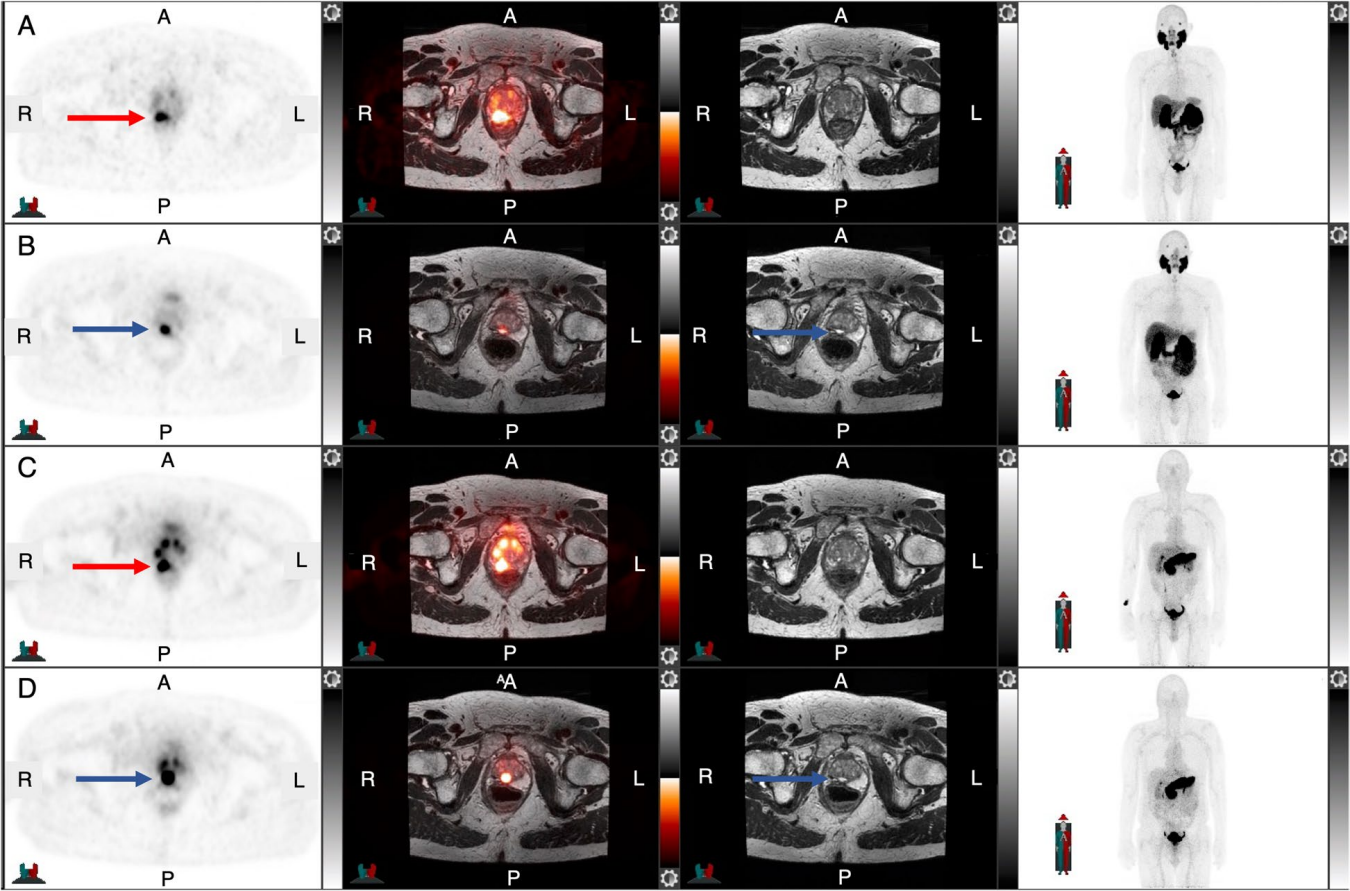
78-year-old man presents with PSA 15.90 ng/mL and PSA density 0.14 ng/mL^2^ for PET-targeted HIFU of a PI-RADS 5 lesion in the right posterior apex. Pre-therapy ^68^ Ga-PSMA11 **A** and ^68^ Ga-RM2 **C** axial PET, axial fused PET/MRI, MRI, and MIP images show concordant focal uptake in the right posterior prostate (red arrows). After HIFU, ^68^ Ga-PSMA11 **B** and ^68^ Ga-RM2 **D** axial PET, axial fused PET/MRI, MRI, and MIP show structural changes with urine pooling in the treated area (blue arrows) but no pathologic uptake. Biopsy confirmed no evidence of PC in the treated area

An interim analysis of a prospective study evaluated 10 men 3 months after HIFU treatment with ^68^ Ga-PSMA11 PET/MRI [105]. Recurrent disease was seen in 60% which was missed by mpMRI. Our group evaluated the feasibility of a combined approach of ^68^ Ga-PSMA11 and ^68^ Ga-RM2 PET/MRI for HIFU guidance and treatment success evaluation (NCT03949517). The preliminary results show that both ^68^ Ga-PSMA11 and ^68^ Ga-RM2 PET/MRI identified target tumors in 100% and 86%, respectively, and accurately verified response to treatment (Fig. 3). This suggests that molecular imaging might be an useful and noninvasive tool for guidance of HIFU and treatment response assessment. In patients requiring repeat focal treatment, it could be used to assist treatment planning and bypass limitations of post-treatment alterations on mpMRI.

Theragnostic involves molecular-targeted imaging and treatment, and is the epitome of targeted, personalized medicine. A focal theragnostic approach for localized PC has been explored preclinically based on photodynamic therapy. Photodynamic therapy is vascular-based and consists of two parts: one is the intravenous injection of a photosensitizer, which is pharmacologically inactive until exposed to the second part, which is its activation by light through transperineally inserted probes under TRUS-guidance. The activated photosensitizer transfers energy to oxygen leading to the generation of superoxide and hydroxyl free radicals, subsequently resulting in vascular thrombosis and coagulative necrosis [106]. As a vascular photosensitizer is cleared rapidly in the blood stream, it requires multiple cycles of treatment, and uptake by surrounding healthy tissue has been a challenge. Consequently, research focused on the development of tissue-targeted photosensitizers, in particular, a conjugate of a PSMA inhibitor and photosensitizer which, after light activation, led to the desired apoptosis in tumor cells in vitro [107–109]. First in vivo studies in mice showed uptake in PSMA expressing tumor cells following systemic injection of the conjugate, and tumor growth inhibition within 1 week after exposure to light [110], and a decrease in tumor size after 2 days [111]. This concept has not been evaluated clinically but the promising preclinical results warrant further research in the arena of focal theragnostics for localized PC.

The VISION trial has shown impressive results in men with metastatic castration-resistant PC undergoing systematic treatment with lutetium-177 (^177^Lu)-PSMA617 with significantly longer radiographic progression free survival (PFS) compared to standard of care treatment alone [112]. The efficacy of ^177^Lu-PSMA617 in men with localized or locoregional advanced PC is now evaluated in the LuTectomy trial (NCT04430192). One or two cycles of ^177^Lu-PSMA617 is given prior to prostatectomy and lymph node dissection to assess tumor absorbed doses in the prostate and any lymph node metastases. The results of this clinical trial might change the place of targeted radionuclide therapy in the treatment sequence of advanced PC.

## Conclusion

Imaging with mpMRI and subsequent mpMRI-targeted biopsy have significantly improved detection of aggressive PC. Real-time in-bore image guided biopsy showed best yield in clinically significant cancers, however, also require the most resources. Given the wider availability, practicability and lower costs, MR-TRUS fusion-targeted biopsy has become the most commonly used technique. PET/CT- or PET/MRI-targeted biopsy adds value to cases with prior negative mpMRI and/or biopsy.

In localized, nonmetastatic PC, focal therapy of the index tumor has become popular. These include transrectal, transperineal or transurethral HIFU, FLA, and cryotherapy. In-bore targeted focal therapy leverages MR thermometry with real-time heat-modulation and better treatment control. Compared to traditional whole gland treatment, significantly lower functional side effects were observed with this minimal invasive approach. All show early tumor control with each modality having different advantages and disadvantages, may it be quality of image guidance, degree of tissue destruction, or extent of ablation margin. The high local relapse rate may reflect these limitations. Subsequent repeat focal therapy or salvage whole gland treatment are feasible. Thus, initial local approaches might prolong the time to radical whole gland treatment. Prospective long-term oncologic and functional outcome data are still scarce, especially no randomized controlled trials comparing the various focal treatments to each other are yet available. However, the current data are encouraging and further studies are warranted.

Assessment of treatment response is an area of unmet clinical need. PSA decreases after treatment to a variable nadir after focal therapies, and mpMRI is limited by post-treatment artifacts that can mask in-field recurrence. Currently, post-treatment biopsy is the most accurate method for treatment verification. PET might be a suitable non-invasive modality to show treatment success. More prospective studies are needed to support the encouraging preliminary results.

Future developments include artificial intelligence and radiomics assisted risk prediction and treatment planning [113]. The use of robotic arms to support navigation and carrying out biopsy or local treatment may increase precision. Furthermore, advancement in scanner hardware and software will allow for faster MRI sequences and increased image quality. Last and most importantly, with more prospective intermediate- and long-term data, consensus guidelines for focal treatments are needed. These should address the most burning questions of whom to treat with which modality in this growing field.

## Data Availability

Not applicable.

## Abbreviations

bpMRI: Biparametric MRI
CT: Computed Tomography
DCE: Dynamic Contrast-Enhanced
DWI: Diffusion Weighted Images
^18^F: Fluorine-18
FLA: Focal Laser Ablation
^68^Ga: Gallium-68
GRPR: Gastrin Releasing Peptide Receptor
HIFU: High Intensity Focused Ultrasound
^177^Lu: Lutetium-177
mpMRI: Multiparametric Magnetic Resonance Imaging
MRI: Magnetic Resonance Imaging
NCCN: National Comprehensive Cancer Network
PC: Prostate Cancer
PET: Positron Emission Tomography
PFS: Progression Free Survival
PI-RADS: Prostate Imaging Reporting and Data System
PSA: Prostate Specific Antigen
PSMA: Prostate Specific Membrane Antigen
T2WI: T2 Weighted Imaging
TRUS: Transrectal Ultrasound
TULSA: Transurethral Ultrasound Ablation
